# The Trend in the Prevalence of Diabetes Mellitus in the Mexican Indigenous Population From 2000 to 2018

**DOI:** 10.1016/j.focus.2023.100087

**Published:** 2023-02-21

**Authors:** Lilia V. Castro-Porras, Rosalba Rojas-Martínez, Martín Romero-Martínez, Carlos A. Aguilar-Salinas, Consuelo Escamilla-Nuñez

**Affiliations:** 1 Policies, Population and Health Research Center, Faculty of Medicine, Universidad Nacional Autónoma de México, Mexico City, Mexico; 2 Reproductive Health Department, Center for Population Health, Instituto Nacional de Salud Publica, Mexico City, Mexico; 3 Center for Evaluation and Survey Research, Instituto Nacional de Salud Publica, Mexico City, Mexico; 4 Metabolic Diseases Research Unit, Instituto Nacional de Ciencias Medicas y Nutricion Salvador Zubiran, Mexico City, Mexico; 5 Department of Endocrinology and Metabolism, Instituto Nacional de Ciencias Medicas y Nutrición Salvador Zubiran, Mexico City, Mexico; 6 Tec Salud, Instituto Tecnológico y de Estudios Superiores de Monterrey, Monterrey, Mexico; 7 Environmental Health Department, Center for Population Health, Instituto Nacional de Salud Publica, Mexico City, Mexico

**Keywords:** Diabetes, indigenous population, trends, prevalence, population-based survey, Mexico

## Abstract

**Introduction:**

Diabetes is a worldwide public health problem. In Mexico, diabetes was the third leading cause of death in the total population in 2020. The indigenous people in Mexico are approximately 6%. This study aims to estimate the trends in diabetes prevalence from 2000 to 2018 in the group of Mexican indigenous language speakers and to analyze the main sociodemographic (e.g., age, educational and socioeconomic level, and the urbanicity of the area of residence) and clinical (e.g., age of diabetes onset, years with diabetes, and BMI) characteristics of this group.

**Methods:**

This cross-sectional study included participants aged ≥20 years from 4 National Health Surveys, 2000–2018. We presented the analyses for indigenous and nonindigenous strata. Logistic models adjusted were used to estimate the trend of diabetes in the study period.

**Results:**

We found a significant increase in the prevalence of diabetes in the indigenous group. This trend in the ORs was maintained when adjusting for age, sex, waist circumference, and area of residence. For the study period, the prevalence change in diagnosed diabetes in the indigenous group was greater than that in the nonindigenous group (OR=6.4, 95% CI=4.1, 8.8 and OR=3.3, 95% CI=2.5, 4.1, respectively). We also found a significant prevalence change in undiagnosed diabetes for the indigenous group (OR=7.7, 95% CI=1.3, 14.6).

**Conclusions:**

In contrast to the results in nonindigenous populations, our main result reveals an increasing probability of being diabetic in the indigenous population from 2006 to 2018. It is necessary to clarify the origin of the accelerated change in diabetes prevalence among the indigenous population in Mexico.

## HIGHLIGHTS

• Mexican indigenous people are more likely to be diabetic than nonindigenous people.
• Since 2006, indigenous people have experienced an increase in obesity.
• Since 2006, indigenous people have experienced an increase in abdominal obesity.
• Indigenous students had lower education scores than nonindigenous students.

## INTRODUCTION

Diabetes is a global public health problem; it has been estimated that approximately 463 million (9.3%) adults aged 20–79 years were living with diabetes worldwide in 2019, and this number is expected to increase to approximately 578 million (10.2%) by 2030 and 700 million (10.9%) by 2045. An estimated 4.2 million deaths among adults in the same age group are attributable to diabetes. Globally, diabetes is estimated to contribute to 11.3% of deaths.^1^

It was recently reported that 10.3% of Mexican adults had been diagnosed with type 2 diabetes (T2D).^2^ A significant number of people living with diabetes do not know their condition; data from the Encuesta Nacional de Salud y Nutrición 2006 showed that 7.07% of Mexicans did not know that they had diabetes.^3^ Diabetes has been associated with heart failure,^4^ rheumatic diseases,^5^ musculoskeletal disorders,^6^ increased risk of fracture,^7^ and colorectal cancer,^8^ among many other negative health-related outcomes.

The increase in energy density of Mexican eating patterns^9^ and sedentary lifestyles^10^ have been identified as the most important causes of diabetes. Both causes are often associated with the biological inability to adapt to aspects of urban culture.^11^

Mexico has seen an increase in the number of metropolitan areas in recent years.^12^ However, there is still a significant percentage of the population living in areas with <2,500 inhabitants.^13^ The traditional areas where the indigenous population is concentrated are located in the most rugged areas of the country, with the most difficult access and deficient communication systems. Nevertheless, the indigenous population is present in almost all municipalities and in almost all the country's entities.^14^ In 2020, there were 7,364,645 indigenous people aged ≥3 years speaking an indigenous language in Mexico, representing 6% of the total population.^15^

Indigenous people, representing 5% of the world's population and 10% of the world's poorest people, continue to be disproportionately poorer, less educated, and in worse health conditions than nonindigenous groups.^16^ Disparities in Mexico's indigenous population in terms of basic and crucial development indicators have been shown; although development indicators have improved for the indigenous population, when we compare indigenous with nonindigenous people, the gap in socioeconomic and development indicators persists.^17^

In addition, it has been reported that the prevalence of diabetes by previous medical diagnosis has been higher in urban localities than in rural localities; however, the difference has shortened over time, reported to be 9.5% vs 9.2% in 2016, respectively.^18^ It is noteworthy that a high rural component is located in the south of Mexico and is the region where many indigenous language speakers are concentrated.^19^

This study aims to estimate the trends in the prevalence of diabetes from 2000 to 2018 in the Mexican indigenous language–speaking population and to analyze the main sociodemographic and clinical characteristics of this population group. We hypothesized that in recent years, the change in the prevalence of diagnosed diabetes in the indigenous population is greater than in the nonindigenous population.

## METHODS

### Study Population

We used data set from the National Health Survey 2000 and the National Health and Nutrition Surveys 2006, 2012, and 2018. All of these are national, cross-sectional, and population-based surveys. For this report, we included the information of people aged ≥20 years with a complete questionnaire and biochemical analyses. All surveys were stratified, and probability sampling was used with national representation. We used the linguistic perspective as a proxy for indigenism. We classified an individual as indigenous if he or she responded affirmatively to the question, (*NOMBRE) habla alguna lengua indígena (dialecto)? Does she/he speak any indigenous language?,* making a distinction between people who speak an indigenous language and those who speak only Spanish (no/yes).

### Measures

We considered that an individual had a previous diagnosis of diabetes if he/she answered yes to the questionnaire question, *Has any doctor told you that you have diabetes?* We considered an individual to have undiagnosed diabetes if they answered *NO* to the previous question and had a fasting serum glucose ≥126 mg/dL with at least 8 hours of fasting or had HbA1c levels ≥6.5%. All measurements were performed under standard procedures.

Table 1 was built to show the general characteristics of the study population. Age was categorized into 3 groups: 20–39, 40–59, and ≥60 years. For educational level, 4 categories were considered: elementary school or less, junior high school, high school, and bachelor's degree or more. The social security variable was divided into 2 categories: no (no security category) and yes (any security category: Instituto Mexicano del Seguro Social, Instituto de Seguridad y Servicios Sociales de Los Trabajadores del Estado, private insurance, and Seguro Popular [as of 2020, it has been replaced by the Instituto Nacional de Salud para el Bienestar, and others: Pemex, Defensa Nacional, Marina Nacional]). For the region, the same classification as the one applied in the surveys of this study was used: North (Baja California, Baja California Sur, Coahuila, Chihuahua, Durango, Nuevo León, Sonora, Tamaulipas), Center (Aguascalientes, Colima, MEX, Guanajuato, Jalisco, Michoacán, Morelos, Nayarit, Querétaro, San Luis Potosí, Sinaloa, Zacatecas, and Mexico City), and South (Campeche, Chiapas, Guerrero, Hidalgo, Oaxaca, Puebla, Quintana Roo, Tabasco, Tlaxcala, Veracruz, Yucatán). Finally, regarding the urbanicity of the area of residence, 2 categories were considered: rural (<2,500 inhabitants) and urban (≥ 2,500 inhabitants).^13^

**Table 1:** Population According to Sociodemographic Characteristics in Mexican Adults From 2000 to 2018, by Indigenous Group

	Nonindigenous				Indigenous			
	2000,	2006,	2012,	2018,	2000,	2006,	2012,	2018,
Characteristics	% (95% CI)	% (95% CI)	% (95% CI)	% (95% CI)	% (95% CI)	% (95% CI)	% (95% CI)	% (95% CI)
Frequency in thousands, *n*	47,806	45,762	59,973	77,652	3,496	3,122	4,325	5,062
Sample size observations, *n*	41,245	5,328	8,610	11,985	3,776	501	1,122	1,177
Women	53.1 (52.3, 53.9)	53.5 (51.3, 55.7)	53.6 (51.4, 55.8)	54.6 (53.0, 56.1)	51.4 (49.1, 53.7)	61.8 (55.9, 67.4)	49.0 (42.5, 55.6)	55.8 (51.6, 59.9)
Age, years								
20–39	61.1 (60.3, 61.9)	53.1 (51.0, 55.2)	51.8 (49.5, 54.1)	43.3 (41.7, 45.0)	54.8 (53.0, 56.7)	47.6 (41.8, 53.4)	44.9 (38.4, 51.6)	38.8 (34.8, 43.0)
40–59	26.8 (26.1, 27.5)	32.2 (30.3, 34.1)	33.2 (31.0, 35.4)	36.9 (35.5, 38.4)	31.0 (29.1, 33.0)	33.3 (28.1, 38.8)	35.9 (30.7, 41.6)	37.6 (33.5, 41.9)
≥60	12.1 (11.6, 12.6)	14.7 (13.3, 16.1)	15.0 (13.6, 16.5)	19.8 (18.5, 21.1)	14.2 (12.7, 15.8)	19.2 (15.2, 23.8)	19.1 (15.6, 23.2)	23.6 (20.3, 27.3)
Educational level								
Elementary or less	44.9 (41.5, 48.3)	47.8 (45.6, 50.1)	39.9 (37.7, 42.1)	29.3 (28.0, 30.7)	77.3 (74.1, 80.3)	82.1 (77.4, 85.9)	75.5 (70.6, 79.9)	65.0 (60.5, 69.3)
Secondary	23.8 (23.0, 24.7)	23.9 (22.2, 25.8)	28.5 (26.4, 30.6)	27.0 (25.6, 28.4)	13.1 (11.3, 15.1)	11.6 (8.3, 16.0)	14.1 (11.3, 17.4)	21.7 (18.5, 25.2)
High school	20.7 (18.9, 22.5)	17.1 (15.4, 18.8)	18.6 (17.0, 20.4)	24.9 (23.5, 26.4)	7.1 (5.7, 8.7)	4.4 (2.6, 7.5)	6.6 (4.4, 9.9)	8.2 (6.1, 10.9)
Bachelor or more	10.6 (9.4, 12.0)	11.1 (9.5, 13.0)	13.0 (11.3, 14.9)	18.8 (17.5, 20.2)	2.5 (1.7, 3.8)	1.9 (0.6, 5.2)	3.8 (2.4, 5.9)	5.1 (3.3, 7.8)
Currently working	50.5 (49.5, 51.4)	53.1 (51.1, 55.1)	54.9 (52.7, 57.1)	61.6 (60.1, 63.2)	51.2 (48.7, 53.7)	45.5 (40.0, 51.1)	52.8 (46.5, 59.0)	62.5 (58.8, 66.0)
Social security	97.6 (97.4, 97.7)	51.6 (49.2, 54.0)	75.9 (74.0, 77.8)	82.8 (81.5, 84.0)	96.7 (95.9, 97.4)	33.0 (26.5, 40.2)	73.6 (65.1, 80.7)	87.8 (84.9, 90.2)
Socioeconomic level in terciles								
Tertile 1	27.9 (23.7, 32.7)	25.8 (23.8, 27.9)	22.3 (20.6, 24.1)	26.6 (25.2, 28.1)	79.0 (74.4, 82.9)	74.9 (68.1, 80.6)	66.5 (58.2, 73.9)	74.3 (69.7, 78.4)
Tertile 2	33.2 (32.4, 34.1)	32.6 (30.4, 34.9)	33.3 (31.3, 35.4)	33.3 (31.8, 34.8)	15.9 (13.3, 18.9)	16.8 (12.5, 22.1)	20.1 (15.7, 25.3)	19.9 (16.4, 23.9)
Tertile 3	38.9 (34.2, 43.7)	41.6 (38.9, 44.3)	44.4 (42.0, 46.8)	40.1 (38.3, 41.9)	5.2 (3.6, 7.3)	8.4 (4.6, 14.6)	13.4 (6.9, 24.3)	5.8 (4.1, 8.2)
Region								
North	21.2 (20.0, 22.4)	21.7 (19.5, 24.0)	21.3 (19.5, 23.1)	21.4 (20.1, 22.8)	3.0 (2.3, 4.0)	3.0 (1.5, 5.9)	4.4 (2.7, 7.3)	4.6 (3.1, 7.0)
Central	53.0 (51.5, 54.4)	51.4 (48.2, 54.6)	51.8 (49.0, 54.6)	51.0 (48.9, 53.1)	10.2 (8.5, 12.3)	14.5 (8.9, 22.8)	20.6 (13.0, 31.0)	20.1 (13.2, 29.2)
South	25.9 (23.4, 28.5)	26.9 (24.5, 29.5)	26.9 (24.7, 29.3)	27.6 (25.8, 29.4)	86.7 (84.1, 88.9)	82.4 (74.3, 88.4)	75.0 (65.2, 82.8)	75.3 (66.5, 82.4)
Area of residence								
Rural	18.7 (13.2, 25.8)	15.0 (13.0, 17.1)	19.1 (17.1, 21.3)	19.7 (17.8, 21.7)	57.8 (51.9, 63.5)	53.2 (43.7, 62.4)	57.9 (48.9, 66.4)	49.8 (41.5, 58.0)
Urban	81.3 (74.2, 86.8)	85.0 (82.9, 87.0)	80.9 (78.7, 82.9)	80.3 (78.3, 82.2)	42.2 (36.5, 48.1)	46.8 (37.6, 56.3)	42.1 (33.6, 51.1)	50.2 (42.0, 58.5)

A descriptive analysis was performed to present an overview of the disease in this population, including people with diabetes (both previously diagnosed by a physician and those who were not). Table 2 shows the distribution (frequencies and 95% CIs) of diabetes by health characteristics stratified by the indigeneity group for each survey. We estimated the age at diabetes onset by subtracting the time of diabetes diagnosis from the current age. The former information was obtained from the questionnaire question, *How long ago were you told you had diabetes?*

**Table 2:** Change in the Diabetes Prevalence in Mexican Adults by Indigenous Group and Year of Survey

	Diagnosed diabetes				Undiagnosed diabetes		Total diabetes	
Group	Frequency in thousands,	Sample observations,	Unadjusted prevalence,	Adjusted prevalence,^a^	Unadjusted prevalence,	Adjusted prevalence,^a^	Unadjusted prevalence,	Adjusted prevalence,^a^
	*n*	*n*	% (95% CI)	% (95% CI)	% (95% CI)	% (95% CI)	% (95% CI)	% (95% CI)
Nonindigenous								
2000	46,030	40,194	5.9 (5.5, 6.2)	7.7 (7.3, 8.2)	NA	NA	5.9 (5.5, 6.2)	7.7 (7.3, 8.2)
2006	47,559	5,496	7.5 (6.4, 8.7)	8.7 (7.5, 10.0)	8.8 (7.6, 10.1)	9.5 (8.3, 10.8)	16.9 (15.4, 18.5)	18.3 (16.8, 20.0)
2012	59,973	8,610	8.8 (7.7, 10.1)	9.7 (8.5, 11.0)	4.5 (3.7, 5.5)	4.5 (3.9, 5.3)	13.3 (11.9, 14.9)	14.4 (13.0, 16.0)
2018	77,652	11,985	10.3 (9.4, 11.1)	9.8 (9.1, 10.6)	6.3 (5.3, 7.3)	6.0 (5.2, 6.9)	16.5 (15.3, 17.8)	17.2 (16.1, 18.3)
Change (%)			**3.3 (2.5, 4.1)**		**–2.8 (–4.9, –0.6)**		0.1 (**–**1.1, 1.4)	
Indigenous								
2000	3,357	3,645	3.5 (2.7, 4.5)	4.1 (3.1, 5.2)	NA	NA	3.5 (2.7, 4.5)	4.1 (3.1, 5.2)
2006	3,230	513	5.3 (3.5, 7.8)	5.4 (3.6, 7.9)	4.0 (2.4, 6.6)	3.9 (2.3, 6.5)	9.6 (6.9, 13.1)	9.4 (6.8, 12.9)
2012	4,325	1,122	8.9 (5.6, 13.9)	8.2 (5.6, 11.9)	4.8 (3.3, 6.8)	4.6 (3.3, 6.5)	13.7 (9.9, 18.5)	12.7 (9.8, 16.3)
2018	5,062	1,177	10.3 (8.1, 13.1)	9.2 (7.3, 11.5)	8.5 (5.7, 12.6)	8.2 (5.5, 12.1)	18.8 (14.7, 23.7)	18.7 (15.0, 23.2)
Change (%)			**6.4 (4.1, 8.8)**		**7.7 (1.3, 14.6)**		**6.7 (2.6, 11.0)**	
National								
2000	49,707	44,107	5.7 (5.3, 6.1)	7.4 (7.0, 7.9)	NA	NA	5.7 (5.3, 6.1)	7.4 (7.0, 7.9)
2006	50,794	6,011	7.6 (6.6, 8.8)	8.4 (7.4, 9.7)	8.5 (7.4, 9.7)	9.1 (8.0, 10.3)	16.4 (15.0, 18.0)	17.7 (16.3, 19.3)
2012	64,298	9,732	8.8 (7.7, 10.0)	9.5 (8.4, 10.8)	4.5 (3.7, 5.5)	4.5 (3.9, 5.2)	13.3 (12.0, 14.8)	14.3 (12.9, 15.7)
2018	82,714	13,162	10.3 (9.5, 11.1)	9.7 (9.0, 10.5)	6.4 (5.5, 7.4)	6.2 (5.4, 7.1)	16.7 (15.5, 17.9)	17.3 (16.2, 18.4)
Change (%)			**3.5 (2.7, 4.2)**		**–2.2 (–4.2, –0.1)**		0.5 (**–**0.7, 1.8)	

*Note:* Boldface indicates statistical significance (*p*<0.05).

a Age adjusted using the world population in 2010. ENSA, Encuesta Nacional de Salud; ENSANUT, Encuesta Nacional de Salud y Nutrición; NA, not available.

The mean weight in kilograms, stature in meters, and waist circumference in centimeters by sex were reported. BMI was calculated as a person's weight in kilograms divided by the square of the person's height in meters (kg/m^2^). Then, it was categorized using the WHO criteria (BMI: underweight, <18.5 kg/m^2^; normal, 18.5–24.9 kg/m^2^; overweight, 25–29.9 kg/m^2^; and obese, ≥30 kg/m^2^).^20^ Underweight and normal weight categories were grouped for analysis. Frequency for each BMI category was reported.

We used the cut off point of waist circumference >102 cm for men and >88 cm for women to define abdominal obesity and then estimated its proportion. To estimate the frequency of hypertriglyceridemia, hypertension, chronic kidney disease, previous stroke, chronic heart failure, cerebral vascular disease, and depression, we considered the questionnaire question that inquired about the presence of this condition in the past or present, depending on the event asked.

Finally, participants were considered current smokers if they answered affirmatively to one of the questions in the questionnaire: *do you currently smoke tobacco every day? Do you currently smoke tobacco some days?* They were considered current drinkers if they answered affirmatively to the question, *do you currently drink?*

### Statistical Analysis

To describe the study population, nonindigenous and indigenous, according to socioeconomic characteristics, we obtained means and percentages with their CIs, considering the variable type (quantitative or qualitative). Change in the prevalence of diabetes (i.e., diagnosed, undiagnosed, and total diabetes) by indigenous group, national level, and year of the survey was obtained with the coefficient from a logistic regression model. We also calculated the prevalence adjusted for age using the world population in 2010 by the direct method.^21^

In addition, we presented health conditions related to diabetes and lifestyle habits with means and percentages with CIs by the indigenous/nonindigenous group. Finally, to estimate diabetes prevalence trends from 2000 to 2018 for each indigenous/nonindigenous group, we used multiple logistic regression models to adjust the risk of diabetes by year of the survey. Both models were adjusted for sex, age, waist circumference, and urbanicity of the area of residence. In the same way, we presented a graph with the predicted probabilities of having diabetes by indigenous group. Previously, bivariate models were constructed; the diagnosis of diabetes and the year of the survey were fixed, and then each variable was included one by one in the models to identify possible confounding factors. The variables considered were those with *p*<0.20 in the bivariate analysis.

The goodness of fit of the models was assessed with the Hosmer–Lemeshow test. We considered a significance level of *p*<0.05 and a 95% CI in all cases. For all analyses, we considered the effect of the complex sample design, using the survey module (svy) of the Stata software, Version 15 (Stata Corp, College Station, Texas).

### Ethical Considerations

All surveys considered in this report were approved by the Ethics, Research, and Biosafety Commissions of the National Institute of Public Health. Participants signed an informed consent form after the survey procedures were explained to them.^22^, ^23^, ^24^, ^25^

## RESULTS

This study included 73,744 participants (45,021; 5,829; 9,732; and 13,162 for 2000, 2006, 2012, and 2018 National Surveys considered, respectively). This sample represented 247.2 million people aged ≥20 years for the whole period. The indigenous stratum had 6,576 participants, representing 16.0 million Mexican adults.

The nonindigenous group had a lower frequency of adults aged ≥60 years than the indigenous group for all surveys (12.1% vs 14.2%, 14.7% vs 19.2%, 15.0% vs 19.1%, and 19.8% vs 23.6% for 2000, 2006, 2012, and 2018 surveys, respectively) (Table 1).

Regarding educational level, for both the nonindigenous and indigenous groups, the category of primary or less showed the highest frequencies; however, there was a wide difference between them (44.9% vs 77.3%, 47.8% vs 82.1%, 39.9% vs 75.5%, and 29.3% vs 65.0% for 2000, 2006, 2012, and 2018 surveys, respectively).

For the nonindigenous group, the third tertile of socioeconomic level reported the highest frequency (38.9%, 41.6%, 44.4%, and 40.1% for 2000, 2006, 2012, and 2018 surveys, respectively); in contrast, for the indigenous group, the first tertile presented the highest prevalence (79.0%, 74.9%, 66.5%, and 74.3% for 2000, 2006, 2012, and 2018 surveys, respectively).

The central region presented the highest concentration of nonindigenous population (53.0%, 51.4%, 51.8%, and 51.0% for 2000, 2006, 2012, and 2018 surveys, respectively), whereas the southern region had the highest concentration of indigenous population (86.7%, 82.4%, 75.0%, and 75.3% for 2000, 2006, 2012, and 2018 surveys, respectively).

The largest nonindigenous population was found in the urban areas of residence (81.3%, 85.0%, 80.9%, and 80.3% for 2000, 2006, 2012, and 2018 surveys, respectively); conversely, the highest concentration of indigenous population was found in the rural areas of residence (57.8%, 53.2%, 57.9%, and 49.8% for 2000, 2006, 2012, and 2018 surveys, respectively) (Table 1).

Table 2 reports the change in the prevalence of diagnosed diabetes, undiagnosed diabetes, and total diabetes for the nonindigenous, indigenous, and the entire population. A positive and significant prevalence change was found for diagnosed diabetes for the whole population and for the nonindigenous and indigenous groups (3.5%, 3.3%, and 6.4%, respectively). In the case of undiagnosed diabetes, a significant negative percentage change was observed for the nonindigenous group and for the total population (–2.8% and –2.2%, respectively). In contrast, for the indigenous group, it was significantly positive (7.7%). Data from the National Health Survey 2000 survey were not available to estimate the prevalence of undiagnosed diabetes.

In Table 3, a total of 8,992 participants living with diabetes were considered: 2,940 (2,778+162), 2,252 (2,067+185), 1,415 (1,258+157), and 2,385 (2,155+230) for the 2000, 2006, 2012, and 2018 National Surveys, respectively. This represented 28.4 million people with diabetes aged ≥20 years for the whole period. The indigenous stratum had 734 participants in the period, representing 1.9 million indigenous adults with diabetes.

**Table 3:** Health Characteristics in Mexican Adults With Diabetes by Indigenous Group and Year of Survey

	2000,	2006,	2012,	2018,	*p* trend	2000,	2006,	2012,	2018,	*p* trend
Characteristics	% (95% CI)	% (95% CI)	% (95% CI)	% (95% CI)		% (95% CI)	% (95% CI)	% (95% CI)	% (95% CI)	
	Nonindigenous					Indigenous				
Frequency in thousands, *n*	2,695	3,011	7,980	12,821		117	202	591	953	
Sample observations n	2,778	2,067	1,258	2,155		162	185	157	230	
Characteristics related to diabetes										
Age of diabetes onset, mean	46.5 (45.5, 47.4)	47.4 (46.2, 48.5)	48.4 (45.8, 51.0)	47.1 (46.0, 48.2)		48.6 (46.1, 51.1)	48.0 (44.9, 51.2)	46.9 (45.0, 48.9)	47.5 (44.9, 50.0)	
Years with diabetes, mean	8.8 (8.3, 9.4)	8.4 (7.2, 9.6)	9.9 (8.9, 10.9)	11.7 (10.8, 12.6)	a	8.7 (6.8, 10.6)	6.7 (4.4, 9.1)	8.0 (5.8, 10.2)	8.7 (6.7, 10.7)	
Characteristics related to health condition										
BMI										
Normal	24.9 (22.0, 28.1)	18.8 (14.7, 23.7)	19.1 (14.2, 25.1)	17.5 (14.3, 21.3)		30.7 (22.3, 40.6)	22.7 (12.2, 38.2)	20.3 (12.0, 32.1)	18.3 (13.3, 24.7)	
Overweight	40.3 (37.4, 43.3)	43.3 (38.0, 48.7)	37.6 (32.4, 43.1)	38.2 (34.4, 42.1)	a	56.1 (46.1, 65.6)	37.1 (25.3, 50.8)	41.9 (24.3, 61.8)	29.6 (20.3, 40.8)	a
Obesity	34.7 (31.8, 37.8)	37.9 (32.8, 43.3)	43.3 (37.5, 49.2)	44.3 (40.7, 48.0)	a	13.2 (7.9, 21.2)	40.2 (27.4, 54.4)	37.8 (24.2, 53.7)	52.1 (40.7, 63.4)	a
Abdominal obesity	65.7 (62.8, 68.6)	60.3 (54.6, 65.6)	62.9 (57.2, 68.3)	71.5 (67.3, 75.3)	a	53.5 (40.2, 66.2)	54.8 (40.4, 68.4)	66.9 (52.1, 79.0)	65.7 (58.3, 72.4)	a
Hypertriglyceridemia	23.6 (21.2, 26.2)	8.2 (5.9, 11.3)	NA	29.7 (26.7, 32.9)	a	27.9 (17.7, 41.0)	2.9 (0.7, 11.4)	NA	23.3 (14.7, 34.8)	a
Diagnosed hypertension	37.5 (34.9, 40.2)	29.5 (25.2, 34.2)	40.0 (34.2, 46.1)	36.7 (33.2, 40.4)	a	34.3 (25.0, 44.9)	26.2 (17.0, 38.2)	29.3 (18.2, 43.5)	29.3 (19.9, 40.8)	
Chronic kidney disease	2.9 (2.1, 3.8)	2.4 (1.4, 3.9)	NA	3.1 (1.9, 5.1)	a	0.0 (0.0, 0.3)	0.0 (.,.)	NA	0.8 (0.2, 3.8)	
Previous stroke	NA	2.2 (1.0, 4.8)	5.3 (3.3, 8.2)	3.3 (2.2, 4.8)	a	NA	1.8 (0.3, 12.1)	0.7 (0.1, 3.3)	0.4 (0.1, 1.7)	
Chronic heart failure	NA	1.3 (0.7, 2.2)	4.8 (2.0, 10.9)	2.4 (1.7, 3.5)	a	NA	1.6 (0.2, 10.9)	0.1 (0.0, 0.8)	0.3 (0.1, 1.6)	a
Cerebral vascular disease	NA	0.4 (0.1, 1.0)	5.1 (2.6, 9.9)	7.5 (2.6, 20.3)	a	NA	1.2 (0.2, 8.2)	15.4 (2.9, 52.2)	0.0 (.,.)	
Depression	NA	23.7 (20.0, 27.9)	32.4 (27.5, 37.9)	25.3 (22.5, 28.4)		NA	29.1 (18.0, 43.6)	20.9 (12.4, 33.1)	18.4 (11.9, 27.4)	
Lifestyle habits										
Current smoker	18.0 (15.8, 20.4)	16.1 (12.5, 20.5)	18.4 (13.8, 24.1)	14.8 (12.4, 17.4)	a	13.1 (7.0, 23.3)	9.7 (4.4, 20.1)	7.1 (2.8, 17.0)	2.8 (1.3, 6.0)	
Current drinker	28.6 (26.3, 31.1)	26.1 (23.1, 29.4)	21.2 (18.0, 24.7)	24.1 (21.5, 26.9)		34.9 (25.3, 45.9)	24.6 (16.6, 34.7)	22.0 (13.1, 34.6)	14.1 (9.0, 21.3)	
Current soft drink consumer (ml), mean	NA	193.5 (173.7, 213.3)	263.9 (184.2, 343.6)	269.5 (229.6, 309.4)	a	NA	162.7 (123.3, 202.2)	91.7 (58.3, 125.1)	195.3 (144.5, 246.1)	a

a Linear *p*trend<0.05. NA, not available.

In the case of participants with previously diagnosed diabetes, the age of diabetes onset was <50 years in both groups. The mean time of diabetes diagnosis was slightly greater than 8 years. For the remaining variables, total diabetes was considered. The obesity category showed a significant increasing trend for both groups; however, in the indigenous group, this was higher (13.2%, 40.2%, 37.8%, and 52.1% for 2000, 2006, 2012, and 2018 surveys, respectively). A similar result was found for abdominal obesity (53.25%, 54.8%, 66.9%, and 65.7% for 2000, 2006, 2012, and 2018 surveys, respectively) (Table 3).

**Figure 1:**
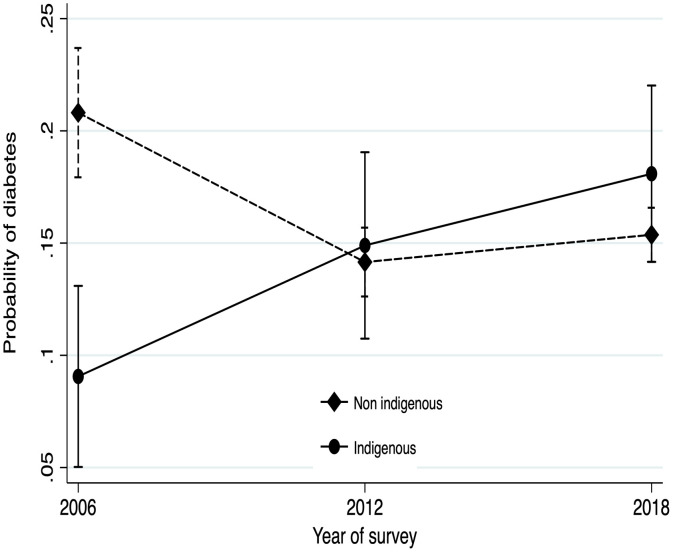
Predicted probabilities by indigenous/nonindigenous group. The models were adjusted for sex, age, waist circumference, and urbanicity.

We found ORs for diabetes trends adjusted for each sociodemographic characteristic or anthropometrics stratified by indigenous group (Appendix, available online); these trends (ORs) were similar when they were simultaneously adjusted for sex, age, waist circumference, and urbanicity. In the indigenous group, we found an increased risk of a person living with diabetes in recent years compared with in the 2006 survey year (OR=1.77, 95% CI=1.03, 3.03 for 2012 and OR=2.22 95% CI=1.35, 3.66 for the 2018 survey years). In contrast, for the nonindigenous group, we found lower risk (OR=0.73 95% CI=0.65, 0.88 for 2012 and OR=0.82 95% CI=0.70, 0.97 for 2018) (Table 4). In addition, the probability of having diabetes from 2006 to 2018 by the indigenous group was observed; a positive trend was found in the indigenous group, whereas a flat trend was observed in the nonindigenous group (Figure 1).

**Table 4:** The Adjusted Risk of Diabetes by Year of the Survey

	Indigenous group		No indigenous group	
Year of survey	OR (95% CI)	*p*-Value	OR (95% CI)	*p*-Value
2006	1		1	
2012	1.77 (1.03, 3.03)	0.038	0.73 (0.61, 0.88)	0.001
2018	2.22 (1.35, 3.66)	0.002	0.82 (0.70, 0.97)	0.017

*Note:* Shown are models for risk of diabetes by year of survey, stratified by indigenous group and adjusted for sex, age, waist circumference, and urbanicity.

## DISCUSSION

The term diabetes describes a group of metabolic disorders characterized and identified by the presence of hyperglycemia in the absence of treatment. The long-term specific effects of diabetes include retinopathy, nephropathy, and neuropathy, among other complications.^26^^,^^27^ Since the beginning of this century, mortality from this cause has been among the top 3 in Mexico; for example, in 2020, the leading cause of death was heart disease, with 220,000 deaths, whereas there were just over 150,000 deaths from diabetes (the third leading cause).^28^

Although the total prevalence of diabetes has remained stable during the last few years, it is possible to observe that undiagnosed diabetes has decreased during the study period. Both diagnosed and undiagnosed diabetes continue to represent a challenge for the health system.

This study is the first to analyze the trend in the prevalence of diabetes among the Mexican indigenous population. We found an increased risk of having diabetes in this group as the survey year increased. In contrast, among the nonindigenous group, we did not find an increased risk, which indicates the differential behavior of the disease between indigenous and nonindigenous groups. T2D is associated with several risk factors, including genetics and the interaction between genetics and environmental factors. On the basis of the findings of previous studies, some of the variants associated with the presentation of T2D in the Mexican mestizo population have been identified. Some of these are related to insulin resistance and insulin secretion. However, the variants found do not explain the high prevalence in the population. Sánchez-Pozos and Menjívar^29^ recommend carrying out additional studies to find new genetic markers for predicting diseases as well as new therapeutic targets. In an analysis of samples from 11 indigenous groups, Granados-Silvestre et al.^30^ identified the T130I polymorphism in the *HNF4A* gene. This polymorphism is associated with the presence of elevated levels of triglycerides and with the early onset of T2D in mestizo Mexicans. In addition, the high frequency of the T130I polymorphism in Mexican indigenous populations reveals a diabetogenic background, which contributes to the high prevalence of T2D in the Mexican population.^30^

In a systematic review, Esparza-Romero and colleagues^31^ reported the prevalence of diabetes mellitus in different indigenous groups in Mexico. The highest prevalence was reported among Mixtecs from Baja California (26.2%) and Yaquis from Sonora (18.3%), and the lowest was reported in Tepehuanos (0.83%) and Mazatecs (2.0%).^31^ Other researchers have obtained a high prevalence of diabetes in indigenous populations of Mexico, such as in San Quintin, Baja California (21.8%) (diagnosed and undiagnosed diabetes included).^32^

Between November 2012 and October 2017, 2,596 Mexican Amerindians from 73 indigenous communities of 60 different ethnic groups participated in a cross-sectional study at Instituto Nacional de Medicina Genómica. This research only included people who identified themselves as indigenous, had parents and grandparents born in the same community, and spoke the native language. Mendoza-Caamal et al.^33^ found that a prevalence of T2D previously diagnosed was 13.7% (95% CI=12.4, 15.1), was 14.3% (95% CI=12.7, 16) in females, and was 12.3% (95% CI=10.1, 14.8) in males. The overall prevalence of elevated fasting glucose (≥100 mg/dL or previous diagnosis of T2D) was 27.9% (95% CI=26.1, 29.6%), with an increase with age—up to 40% in adults aged from 41 years to 70 years—and no difference by sex.^33^ The differences obtained in the previously listed studies were also due to the definition used to determine a person as indigenous—self-identified as indigenous or speaker of an indigenous language—and the definition of diabetes, with diagnosis and without a diagnosis, that is, by the blood glucose result from a blood sample, which were not included by all the studies.

By contrast, the correlation between education and health is well established and well studied; some studies have even estimated the causal effect of education on health.^34^ Because of this, we highlighted that in our study, the indigenous group continues to have the lowest education levels. Although this percentage has decreased by about 10 percentage points during the study period, more is needed. In 2018, more than half of this population was in the lowest educational category (65.0% [60.5, 69.3]), whereas among the nonindigenous population, less than one third were in this category (29.3% [28.0, 30.7]). These results are in line with the socioeconomic situation, where the indigenous population is concentrated in the first tertile for the entire study period. This means that 3 of 4 indigenous adults were in this category. Our results coincide with those reported by Juárez-Ramírez and colleagues,^35^ who conducted a study of 556 Mexican participants with 35% indigenous population; they found that illiteracy was higher in indigenous and rural localities than in urban ones. In addition to lower schooling, other risk factors have been reported that are associated with T2D in indigenous Mexicans, including age; female status; family history of diabetes, obesity, and hypertension; and larger waist/hip ratios.^31^ We found similar results to theirs, except in the case of age, which can be explained by their cross-sectional design, which may have underrepresented older participants because they included 66% of nonprobability samples.^31^

An inverse association between SES and type 2 prevalence had previously been claimed.^36^ However, in our analysis, this association could not be established. We report a lower prevalence of diabetes in the early years of the study among the poorest people, which is the indigenous group. Interestingly, for the last year of the survey, the prevalence in this group was higher than that of the nonindigenous group, suggesting that factors other than SES could affect the prevalence of diabetes more.

According to our results, the trend of obesity among the indigenous group in Mexico has significantly increased from 13.2% in 2000 to 52.1% in 2018; this difference represents about 40 percentage points in 18 years. Although the trend increased in the nonindigenous group, the difference in the same period is about 10 percentage points, going from 34.7% in 2000 to 44.3% in 2018. This trend is consistent with the annualized average of 2.3 percentage points, suggesting that the distribution has shifted to the right with a total increase of 42.2% over the 2000–2018 period.^37^ The percentage change obtained between surveys for diabetes prevalence for the indigenous group was 6.7% (2.6, 11.0) and 0.1% (–1.1, 1.4) for the nonindigenous group. Nationally, the percentage change was 0.5% (–0.7, 1.8). Only the percentage for the indigenous group was statistically significant.

By contrast, more than half of the nonindigenous group presented abdominal obesity during the entire study period. This result was superior to that of Villalta et al.^38^ in a cross-sectional study of 195 indigenous women aged over 45 years; they found that the most frequent component of metabolic syndrome (30%) was abdominal circumference. On the basis of the analysis of another national survey, the first wave (2002) of the Mexican Family Life Survey, a longitudinal study of Mexican households and communities, Stoddard and colleagues^39^ found that indigenous adults had significantly lower odds of obesity (OR=0.58, 95% CI=0.49, 0.68) and diabetes (OR=0.59, 95% CI=0.40, 0.68) than nonindigenous adults, when *indigenous status* was defined as the ability to speak an indigenous language, after adjusting for lifestyle indicators. In this case, *diabetes* was defined only for diagnosed diabetes.^39^

Some authors stated that acculturated societies are entering a dangerous era with rapidly increasing rates of obesity in populations leading to rising levels of T2D^40^^,^^41^; in this sense, Mexico has experienced a change in eating habits in recent decades. Groenendael stated that the most critical change had been the shift from a traditional rural diet of corn and beans to a highly commercialized and industrialized diet under the influence of changing economic models. This change is also observed in rural areas of Mexico,^42^ where the indigenous population is mainly found. Consumption of ultraprocessed food and sugar-sweetened beverages has been linked to an increased risk of obesity and other metabolic disorders.^43^^,^^44^ In a study conducted in communities in Mexico with a predominantly indigenous population, the high consumption of tortillas (15–20 tortillas per day) and the daily, social, and sumptuary consumption of soft drinks and beer have been associated with excessive caloric intake.^45^

Differences in the health lifestyles of indigenous people may be associated with a higher prevalence of obesity; therefore, it is possible to suggest that this change in body composition could be related to the increased prevalence of diabetes. Soto-Estrada et al.^9^ suggested that the steady increase in the mortality rate from T2D from 1990 to 2015 coincides with the increase in the energy density of Mexican dietary patterns from 1961 to 2013. Different dietary patterns are one of several factors involved, some of which could be related to lifestyle changes.

Although this study did not examine diabetes control, Cruz-Sánchez and Cruz-Arceo's findings deserve attention. In an indigenous community in Tabasco, they conducted qualitative research about diabetes. The researchers found that women have access to the health service once a month to receive health education and to control chronic diseases to maintain social inclusion programs. They are diagnosed with diabetes when they already have symptoms or complications. In some cases, peritoneal dialysis or insulin was applied when the damage had progressed to a very advanced level. The abandonment of medical treatment can result in blindness or death in the indigenous population. There is a greater trust in herbal medicine among this group. Diabetes self-care requires daily glucose monitoring, preferably in the morning, as part of self-care. There was no equipment available to any of the study participants. Every month, health center staff perform this examination during control visits. Indigenous communities’ poverty conditions determine this.^46^ Even though Mexican guidelines for diabetes treatment exist, few physicians are familiar with or follow them. In addition, primary care clinics (which treat most cases) lack the infrastructure to treat chronic diseases. Individual consultations are too brief, and other health professionals are rarely included and involved.^47^

### Limitations

Among the strengths of this study is the design that allows the representation of the adult population in Mexico. Using similar methodologies between 2000, 2006, 2012, and 2018 surveys will enable us to assess the trend in diabetes prevalence. Nevertheless, we recognize that using the criterion of speaking an indigenous language to identify the indigenous population may be limited. One of the problems that arises when trying to include possible confounding factors is the lack of precise information on the indigenous group, for example, information about a lifestyle: sweet beverage consumption and sedentarism behavior. Finally, our study is cross-sectional, which limits our ability to generate causal hypotheses.

## CONCLUSIONS

In contrast to the probability among nonindigenous populations, our main result reveals an increased probability of being diabetic among the indigenous population from 2006 to 2018. It is necessary to clarify the origin of the accelerated change in diabetes prevalence among the indigenous population in Mexico.

## Appendix Supplementary materials

Image, application 1

## Footnotes

Supplementary material associated with this article can be found in the online version at doi:10.1016/j.focus.2023.100087.
