# Supplementary material for: The Trend in the Prevalence of Diabetes Mellitus in the Mexican Indigenous Population From 2000 to 2018
Source: AJPM Focus. 2023 Feb 21;2(2):100087. doi: 10.1016/j.focus.2023.100087 (PMC10546564; doi:10.1016/j.focus.2023.100087)
Supplement: Supplementary file 1 [file mmc1.docx]

| **Appendix: ORs^1^ and 95% CIs for diabetes trends adjusted by main sociodemographic characteristics for each indigenous group.** | | | | | | | | | | | | | |
| --- | --- | --- | --- | --- | --- | --- | --- | --- | --- | --- | --- | --- | --- |
|  | | | | | | | | | | | | | |
|  | **Non indigenous population** | | | | | | | | | | | | |
| **Characteristic** |  |  |  |  |  |  |  |  |  |  |  |  |  |
| ***Demographic characteristics*** |  |  |  |  |  |  |  |  |  |  |  |  |  |
| Survey year | Sex | Age (years) | Age categories | Educational level | Living with partner | Working | Social Security | Residential area | Region | Socioeconimic level | BMI | Abdominal obesity | Waist circumference |
| 2012 | 0.63 [0.49,0.80]* | 0.65 [0.52,0.82]* | 0.66 [0.52,0.83]* | 0.71 [0.57,0.88]* | 0.70 [0.57,0.87]* | 0.60 [0.48,0.75]* | 0.63 [0.50,0.79]* | 0.70 [0.56,0.87]* | 0.69 [0.56,0.86]* | 0.66 [0.54,0.82]* | 0.64 [0.52,0.80]* | 0.56 [0.45,0.71]* | 0.71 [0.57,0.89]* |
| 2018 | 0.81 [0.65,1.00]* | 0.76 [0.61,0.94]* | 0.74 [0.60,0.91]* | 0.99 [0.81,1.22] | 0.91 [0.75,1.11] | 0.80 [0.65,0.97]* | 0.79 [0.65,0.97]* | 0.90 [0.74,1.10] | 0.89 [0.73,1.08] | 0.86 [0.71,1.04] | 0.80 [0.65,0.98]* | 0.67 [0.54,0.83]* | 0.85 [0.70,1.04] |
| Sex (women) | 1.19 [1.03,1.37]* |  |  |  |  |  |  |  |  |  |  |  |  |
| Age(year) |  | 1.05 [1.04,1.05]* |  |  |  |  |  |  |  |  |  |  |  |
| Age categories |  |  |  |  |  |  |  |  |  |  |  |  |  |
| 20-39 |  |  | 1 |  |  |  |  |  |  |  |  |  |  |
| 40-59 |  |  | 4.87 [4.00,5.93]* |  |  |  |  |  |  |  |  |  |  |
| 60 or more |  |  | 8.51 [6.90,10.5]* |  |  |  |  |  |  |  |  |  |  |
| Educational level |  |  |  |  |  |  |  |  |  |  |  |  |  |
| Less than elementary |  |  |  | 1 |  |  |  |  |  |  |  |  |  |
| Elementary & Secondary |  |  |  | 0.60 [0.49,0.73]* |  |  |  |  |  |  |  |  |  |
| High school or more |  |  |  | 0.31 [0.25,0.39]* |  |  |  |  |  |  |  |  |  |
| Living with partner |  |  |  |  |  |  |  |  |  |  |  |  |  |
| Without partner |  |  |  |  | 1 |  |  |  |  |  |  |  |  |
| With partner |  |  |  |  | 1.35 [1.16,1.56]* |  |  |  |  |  |  |  |  |
| Working |  |  |  |  |  |  |  |  |  |  |  |  |  |
| No |  |  |  |  |  | 1 |  |  |  |  |  |  |  |
| Yes |  |  |  |  |  | 0.61 [0.53,0.71]* |  |  |  |  |  |  |  |
| Social Security |  |  |  |  |  |  |  |  |  |  |  |  |  |
| No |  |  |  |  |  |  | 1 |  |  |  |  |  |  |
| Yes |  |  |  |  |  |  | 1.43 [1.19,1.71]* |  |  |  |  |  |  |
| Residential area |  |  |  |  |  |  |  |  |  |  |  |  |  |
| Urban |  |  |  |  |  |  |  | 1 |  |  |  |  |  |
| Rural |  |  |  |  |  |  |  | 1.38 [1.21,1.58]* |  |  |  |  |  |
| Region |  |  |  |  |  |  |  |  |  |  |  |  |  |
| North |  |  |  |  |  |  |  |  | 1 |  |  |  |  |
| Central |  |  |  |  |  |  |  |  | 1.13 [0.97,1.31] |  |  |  |  |
| South |  |  |  |  |  |  |  |  | 1.01 [0.87,1.17] |  |  |  |  |
| Socioeconimic level (quintiles) |  |  |  |  |  |  |  |  |  |  |  |  |  |
| 1 |  |  |  |  |  |  |  |  |  | 1 |  |  |  |
| 2 |  |  |  |  |  |  |  |  |  | 1.14 [0.94,1.38] |  |  |  |
| 3 |  |  |  |  |  |  |  |  |  | 1.09 [0.90,1.30] |  |  |  |
| 4 |  |  |  |  |  |  |  |  |  | 1.18 [0.97,1.45] |  |  |  |
| 5 |  |  |  |  |  |  |  |  |  | 1.16 [0.93,1.44] |  |  |  |
| BMI |  |  |  |  |  |  |  |  |  |  |  |  |  |
| Normal |  |  |  |  |  |  |  |  |  |  | 1 |  |  |
| Overweight |  |  |  |  |  |  |  |  |  |  | 1.72 [1.40,2.10]* |  |  |
| Obesity |  |  |  |  |  |  |  |  |  |  | 2.25 [1.84,2.76]* |  |  |
| Abdominal obesity |  |  |  |  |  |  |  |  |  |  |  |  |  |
| No |  |  |  |  |  |  |  |  |  |  |  | 1 |  |
| Yes |  |  |  |  |  |  |  |  |  |  |  | 2.46 [2.12,2.86]* |  |
| Waist circumference (cm) |  |  |  |  |  |  |  |  |  |  |  |  | 1.02 [1.01,1.02]* |
|  |  |  |  |  |  |  |  |  |  |  |  |  |  |
|  | **Indigenous population** | | | | | | | | | | | | |
|  |  |  |  |  |  |  |  |  |  |  |  |  |  |
|  |  |  |  |  |  |  |  |  |  |  |  |  |  |
| Survey year | Sex | Age (years) | Age categories | Educational level | Living with partner | Working | Social Security | Residential area | Region | Socioeconimic level | BMI | Abdominal obesity | Waist circumference |
| 2012 | 1.38 [0.75,2.54] | 1.76 [0.95,3.26] | 1.80 [0.97,3.33] | 1.71 [0.91,3.19] | 1.66 [0.92,3.02] | 1.42 [0.78,2.60] | 1.51 [0.82,2.78] | 1.71 [0.91,3.23] | 1.63 [0.89,2.98] | 1.51 [0.85,2.69] | 1.66 [0.88,3.14] | 1.34 [0.73,2.48] | 1.84 [0.98,3.46] |
| 2018 | 1.97 [1.03,3.77]* | 2.46 [1.39,4.38]* | 2.49 [1.39,4.45]* | 2.57 [1.44,4.62]* | 2.42 [1.34,4.38]* | 2.18 [1.19,3.99]* | 2.13 [1.14,3.98]* | 2.41 [1.35,4.30]* | 2.40 [1.37,4.21]* | 2.28 [1.26,4.14]* | 2.22 [1.25,3.92]* | 1.72 [0.94,3.15] | 2.50 [1.39,4.48]* |
| Sex (women) | 1.46 [0.99,2.16] |  |  |  |  |  |  |  |  |  |  |  |  |
| Age(year) |  | 1.03 [1.03,1.04]* |  |  |  |  |  |  |  |  |  |  |  |
| Age categories |  |  |  |  |  |  |  |  |  |  |  |  |  |
| 20-39 |  |  | 1 |  |  |  |  |  |  |  |  |  |  |
| 40-59 |  |  | 7.59 [4.75,12.11]* |  |  |  |  |  |  |  |  |  |  |
| 60 or more |  |  | 5.56 [3.51,8.82]* |  |  |  |  |  |  |  |  |  |  |
| Educational level |  |  |  |  |  |  |  |  |  |  |  |  |  |
| Less than elementary |  |  |  | 1 |  |  |  |  |  |  |  |  |  |
| Elementary & Secondary |  |  |  | 0.91 [0.59,1.41] |  |  |  |  |  |  |  |  |  |
| High school or more |  |  |  | 0.41 [0.21,0.79]* |  |  |  |  |  |  |  |  |  |
| Living with partner |  |  |  |  |  |  |  |  |  |  |  |  |  |
| Without partner |  |  |  |  | 1 |  |  |  |  |  |  |  |  |
| With partner |  |  |  |  | 0.91 [0.52,1.58] |  |  |  |  |  |  |  |  |
| Working |  |  |  |  |  |  |  |  |  |  |  |  |  |
| No |  |  |  |  |  | 1 |  |  |  |  |  |  |  |
| Yes |  |  |  |  |  | 0.65 [0.44,0.97]* |  |  |  |  |  |  |  |
| Social Security |  |  |  |  |  |  |  |  |  |  |  |  |  |
| No |  |  |  |  |  |  | 1 |  |  |  |  |  |  |
| Yes |  |  |  |  |  |  | 1.30 [0.83,2.04] |  |  |  |  |  |  |
| Residential area |  |  |  |  |  |  |  |  |  |  |  |  |  |
| Urban |  |  |  |  |  |  |  | 1 |  |  |  |  |  |
| Rural |  |  |  |  |  |  |  | 1.74 [1.17,2.58]* |  |  |  |  |  |
| Region |  |  |  |  |  |  |  |  |  |  |  |  |  |
| North |  |  |  |  |  |  |  |  | 1 |  |  |  |  |
| Central |  |  |  |  |  |  |  |  | 3.33 [1.27,8.69]* |  |  |  |  |
| South |  |  |  |  |  |  |  |  | 2.21 [1.08,4.54]* |  |  |  |  |
| Socioeconimic level (quintiles) |  |  |  |  |  |  |  |  |  |  |  |  |  |
| 1 |  |  |  |  |  |  |  |  |  | 1 |  |  |  |
| 2 |  |  |  |  |  |  |  |  |  | 1.40 [0.95,2.05] |  |  |  |
| 3 |  |  |  |  |  |  |  |  |  | 1.57 [0.96,2.57] |  |  |  |
| 4 |  |  |  |  |  |  |  |  |  | 3.20 [1.22,8.35]* |  |  |  |
| 5 |  |  |  |  |  |  |  |  |  | 1.03 [0.33,3.14] |  |  |  |
| BMI |  |  |  |  |  |  |  |  |  |  |  |  |  |
| Normal |  |  |  |  |  |  |  |  |  |  | 1 |  |  |
| Overweight |  |  |  |  |  |  |  |  |  |  | 1.41 [0.88,2.27] |  |  |
| Obesity |  |  |  |  |  |  |  |  |  |  | 4.04 [2.78,5.86]* |  |  |
| Abdominal obesity |  |  |  |  |  |  |  |  |  |  |  |  |  |
| No |  |  |  |  |  |  |  |  |  |  |  | 1 |  |
| Yes |  |  |  |  |  |  |  |  |  |  |  | 3.64 [2.60, 5.10]* |  |
| Waist circumference (cm) |  |  |  |  |  |  |  |  |  |  |  |  | 1.02 [1.01,1.03]* |
| ^1^ Logistic regression model; *p<0.05 |  |  |  |  |  |  |  |  |  |  |  |  |  |
|  |  |  |  |  |  |  |  |  |  |  |  |  |  |
|  |  |  |  |  |  |  |  |  |  |  |  |  |  |
|  |  |  |  |  |  |  |  |  |  |  |  |  |  |
|  |  |  |  |  |  |  |  |  |  |  |  |  |  |
|  |  |  |  |  |  |  |  |  |  |  |  |  |  |
|  |  |  |  |  |  |  |  |  |  |  |  |  |  |
|  |  |  |  |  |  |  |  |  |  |  |  |  |  |
|  |  |  |  |  |  |  |  |  |  |  |  |  |  |
|  |  |  |  |  |  |  |  |  |  |  |  |  |  |
|  |  |  |  |  |  |  |  |  |  |  |  |  |  |
